# PARP inhibitors: current status and implications for anticancer therapeutics

**DOI:** 10.1186/1750-9378-8-46

**Published:** 2013-12-02

**Authors:** Hadi Usmani, Syed Ather Hussain, Asfandyar Sheikh

**Affiliations:** 1 Dow Medical College, Dow University of Health Sciences, Karachi, Pakistan; 2 Pakistan Research Evolution Scientific Society, Baba-e-Urdu Road, Karachi 74200, Pakistan

## Abstract

Poly(ADP-ribose) polymerases (PARPs) comprise of a large family of 17 proteins encoded by various genes which participate in genome maintenance, apoptosis, inflammatory responses and the regulation of gene expression programs. PARP inhibitors, as therapeutic agents, come into play acting on both PARP 1 and PARP 2. These drugs seem to target tumor cells in a moment of vulnerability when they are undergoing DNA repair. In the past few years this class of anti-cancer drug has been discovered to show a promising niche in the clinic.

## Letter to the Editor

Poly(ADP-ribose) polymerases (PARPs, aka diphtheria toxin-like ADP-ribosyltransferases [ARTDs]) comprise of a large family of 17 proteins encoded by various genes which participate in genome maintenance, apoptosis, inflammatory responses and the regulation of gene expression programs [1,2]. Out of these 17 proteins, PARP1 and PARP2 are the only enzymes whose catalytic activity is turned on when there is a single strand break (SSB) in the DNA [3].

PARPs constitutes of four functional domains: a DNA-binding domain, a B domain, an auto-modification domain and a catalytic domain. The DNA binding domain comprises of two zinc fingers and is involved in detecting and resealing DNA breaks [4]. In addition to containing a site which is acted upon by caspases resulting in inactivation of PARP, domain B also has a bipartite nuclear localization signal which is involved in the nuclear homing of PARP1 [5]. The automodification domain has a BRCT motif and is responsible for letting go of the protein from DNA after the catalytic reaction has occurred at the catalytic domain. The catalytic domain is involved in the synthesis of the poly (ADP-ribose) chain.

Following DNA damage, PARP binds to nicotinamide adenine dinucleotide and splits it off at the nicotinamide–ribose bond with polymerization of the ADP-ribose moiety [6]. Repetition of this step results in the synthesis of a multi-branched polymer of ADP-ribose on either nuclear protein acceptors located on chromatin or on the PARP itself [7]. These automodification and heteromodification reactions ensure the longevity of damaged replicating cells because the poly (ADP-ribose) chain attracts the attention of DNA repairing enzymes and scaffolding proteins such as DNA ligase III, DNA polymerase beta and X-ray cross-complementing gene 1 which in turn take part in a process called base excision repair (BER) [8].

Two theories have been proposed by which PARP is thought to induce apoptosis in cells. First, there is the caspase independent pathway in which PARP is excessively stimulated, and the other is the caspase dependent pathway in which PARP is inactivated. In the first pathway, it is believed that PARP1 activation and the subsequent synthesis of PAR polymer serves as a cell death signal which leads to the release of apoptosis-inducing factor (AIF) from the mitochondria into the nucleus [9]. AIF then initiates apoptosis. However, in the other pathway, PARP is rapidly and specifically cleaved by caspase-3, a mammalian ICE-related protease. It is believed that PARP cleavage ensures that all cells undergoing self-destruction turn off protective mechanisms like DNA repair associated with PARP activity [10]. The exact mechanism of these two processes and the intermediate steps involved are not fully understood.

Even without complete apprehension of the mechanisms of the Poly(ADP-ribose) polymerases, there has been significant research towards targeting these proteins in cancer patients. This is where the class of PARP inhibitors as therapeutic agents come into play acting on both PARP 1 and PARP 2. These drugs seem to target tumor cells in a moment of vulnerability when they are undergoing DNA repair [11].

Early clinical phase 1 trials were conducted on breast, ovarian and prostate cancer targeting the BRCA1/2 muations. The rationale for use of PARP inhibitors in these cancers represent an exquisite example of how different pathways cooperate to repair damage [12,13]. BRCA1 and BRCA2 have an essential role in DNA double-strand break repair via homologous recombination, whereas PARP, as mentioned earlier, has a role in BER. It has been shown that BRCA1 or BRCA2 dysfunction sensitizes cells to the PARP inhibition, which subsequently results in chromosomal instability and cell cycle arrest and apoptosis, thereby working synergistically and providing a novel therapeutic option without the need for an exogenous DNA-damaging agent [12,13].

Iniparib (BSI 201) being one of the initial candidates showed serious potential but failed to meet expectations in phase III trails and was eventually disproved as a PARP inhibitor [1]. Olaparib (AZD2281), another PARP inhibitor, produced positive anti-tumor results in cancer patients with BRCA1/2 mutations [14]. Currently there are 8 molecules in clinical trials which follow two distinct pathways of pharmacodynamics. The first is a monotherapy which targets tumor cells that have a defect in the DNA repair mechanisms. The second is a combination therapy with DNA damaging chemotherapeutic treatments thus offering a potentiating effect to cause the cell to undergo apoptosis [1]. Patients evaluated in clinical trials with germline BRCA1/2 mutations, through one of those pathways, have seen a 40% response rate with olaparib in platinum sensitive ovarian cancer [15]. PARPis have also been proven to be the first treatment for BRCA1/2 mutation carrier in prostate cancer patients and have promising anti-tumor activity [16].

It is important to know that BRCA1/2 associated cancers are quite rare and only account for less than 5% of breast-cancer patients so it is crucial to see how PARPis function on other cancers. An interesting match is triple negative breast cancer in which tumors lack expression of the estrogen receptor (ER), progesterone receptor (PR), and human epidermal growth factor receptor 2 (HER2) [11]. In a trial conducted by O’Shaughnessy, triple negative breast cancer patients were given iniparib in conjunction with gemcitabine and carboplatin (chemotherapy doublet) which improved the rate of clinical benefit from 34% to 56% (P = 0.01) and the rate of overall response from 32% to 52% (P = 0.02) [17]. Unfortunately despite recent advances in the clinical evaluation of various PARP inhibitors in triple-negative breast cancer (TNBC) patients, there has been very little anti-tumor progression beyond PARPis [18]. Another example is a trial conducted with rucaparib (PF-01367338, AG014699) in combination with temozolomide for the treatment of metastatic melanoma which showed positive signs of chemopotentiation. Results showed that response rate was 17.4%, median time to progression 3.5 months, median overall survival was 9.9 months, and 36% of patients were progression-free at 6 months [19].

Since PARP inhibitors are blocking the pathway to repair damaged DNA; they require a selective increase in DNA damage to tumor cells compared to normal cells in order to produce an enhanced therapeutic effect by chemotherapy. Thus it would be beneficial if the tumor cells already harbored DNA repair defects such as the case with BRCA1/2 mutations. By blocking both DNA damage repair (DDR) pathways, the first being a tumor germline defect in a non-BER pathway and the second being a PARP inhibited BER pathway, it decreases the chances of the tumor cells to withstand DNA damage. This phenomenon is termed ‘synthetic lethality’ and is very effective in preventing the tumor cells from replicating efficiently. This theory could also be applied to include treatment of tumors with defects in other HR pathway proteins such as PTEN-deficient cells which are sensitive to PARP inhibitors probably due to the involvement in the expression of RAD51, another gene encoding for DNA double strand break repairs [15].

One thing to note is the limitations of PARP inhibitors with regards to ‘PARP trapping’ which occurs during PARP inhibitor treatments. In normal cells the PARP1/2 bind to DNA and then recruit other proteins to do the repairing after which the PARPs are released off the DNA. But the inhibitors trap the PARP in the PARP-DNA complex which makes a lethal PARP poison, so to speak. This phenomenon is more detrimental to the cell than the DNA breaks that occur during normal cell replication. Thus PARP inhibitors should be classified not only in their enzyme inhibition abilities but also according to their potency to trap PARP [20].

In the past few years this class of anti-cancer drug has been discovered to show a promising niche in the clinic. There are some limitations including resistance but that is to be expected with cancer cells [21]. But the amount of research being poured into this exciting new class of anti-tumor therapeutics is bound to produce interesting results. Current researchers should be asking the questions related to development of PARP inhibitors that will enhance DNA damage in tumor cells that lack an intrinsic defect in DDR thus being able to divert the synthetic lethality condition [15]. Hopefully with some answers there will be an improved therapy for patients with cancers that have previously been lacking in treatments.

## Competing interests

The authors declare that no competing interests exist.

## Authors’ contributions

AS conceived the topic. HU and SAH were involved in drafting the initial manuscript. AS critically revised the manuscript. The authors have read and approved the manuscript.

## References

[B1] Papeo G Casale E Montagnoli A Cirla A PARP inhibitors in cancer therapy: an update Expert Opin Ther Pat 2013 23 4 503 514 10.1517/13543776.2013.768615 23379721

[B2] Kim MY Zhang T Kraus WL Poly(ADP-ribosyl)ation by PARP-1: ‘PAR-laying’ NAD + into a nuclear signal Genes Dev 2005 19 17 1951 1967 10.1101/gad.1331805 16140981

[B3] Tentori L Graziani G Chemopotentiation by PARP inhibitors in cancer therapy Pharmacol Res 2005 52 1 25 33 10.1016/j.phrs.2005.02.010 15911331

[B4] Petrucco S Sensing DNA damage by PARP-like fingers Nucleic Acids Res 2003 31 23 6689 6699 10.1093/nar/gkg890 14627802 PMC290258

[B5] Kaufmann SH Desnoyers S Ottaviano Y Davidson NE Poirier GG Specific proteolytic cleavage of poly(ADP-ribose) polymerase: an early marker of chemotherapy-induced apoptosis Cancer Res 1993 53 17 3976 3985 8358726

[B6] Curtin NJ PARP inhibitors and cancer therapy Poly (ADP-ribosyl) ation 2006 United States: Springer 218 233

[B7] D’Amours D Desnoyers S D’Silva I Poirier GG Poly(ADP-ribosyl)ation reactions in the regulation of nuclear functions Biochem J 1999 342 Pt 2 249 268 10455009 PMC1220459

[B8] Ame JC Spenlehauer C de Murcia G The PARP superfamily Bioelectromagnetics 2004 26 8 882 893 10.1002/bies.20085 15273990

[B9] Yu SW Andrabi SA Wang H Kim NS Poirier GG Dawson TM Dawson VL Apoptosis-inducing factor mediates poly(ADP-ribose) (PAR) polymer-induced cell death Proc Natl Acad Sci USA 2006 103 48 18314 18319 10.1073/pnas.0606528103 17116881 PMC1838748

[B10] Wang ZQ Stingl L Morrison C Jantsch M Los M Schulze-Osthoff K Wagner EF PARP is important for genomic stability but dispensable in apoptosis Genes Dev 1997 11 18 2347 2358 10.1101/gad.11.18.2347 9308963 PMC316515

[B11] Carey LA Sharpless NE PARP and cancer–if it’s broke, don’t fix it N Engl J Med 2011 364 3 277 279 10.1056/NEJMe1012546 21208102 PMC3712751

[B12] Farmer H McCabe N Lord CJ Tutt AN Johnson DA Richardson TB Santarosa M Dillon KJ Hickson I Knights C Targeting the DNA repair defect in BRCA mutant cells as a therapeutic strategy Nature 2005 434 7035 917 921 10.1038/nature03445 15829967

[B13] Bryant HE Schultz N Thomas HD Parker KM Flower D Lopez E Kyle S Meuth M Curtin NJ Helleday T Specific killing of BRCA2-deficient tumours with inhibitors of poly(ADP-ribose) polymerase Nature 2005 434 7035 913 917 10.1038/nature03443 15829966

[B14] Fong PC Boss DS Yap TA Tutt A Wu P Mergui-Roelvink M Mortimer P Swaisland H Lau A O’Connor MJ Inhibition of poly(ADP-ribose) polymerase in tumors from BRCA mutation carriers N Engl J Med 2009 361 2 123 134 10.1056/NEJMoa0900212 19553641

[B15] Kummar S Chen A Parchment RE Kinders RJ Ji J Tomaszewski JE Doroshow JH Advances in using PARP inhibitors to treat cancer BMC Med 2012 10 25 10.1186/1741-7015-10-25 22401667 PMC3312820

[B16] Sandhu SK Omlin A Hylands L Miranda S Barber LJ Riisnaes R Reid AH Attard G Chen L Kozarewa I Poly (ADP-ribose) polymerase (PARP) inhibitors for the treatment of advanced germline BRCA2 mutant prostate cancer Ann Oncol 2013 24 5 1416 1418 10.1093/annonc/mdt074 23524863

[B17] O’Shaughnessy J Osborne C Pippen JE Yoffe M Patt D Rocha C Koo IC Sherman BM Bradley C Iniparib plus chemotherapy in metastatic triple-negative breast cancer N Engl J Med 2011 364 3 205 214 10.1056/NEJMoa1011418 21208101

[B18] Chuang HC Kapuriya N Kulp SK Chen CS Shapiro CL Differential anti-proliferative activities of poly(ADP-ribose) polymerase (PARP) inhibitors in triple-negative breast cancer cells Breast Cancer Res Treat 2012 134 2 649 659 10.1007/s10549-012-2106-5 22678161 PMC4297209

[B19] Plummer R Lorigan P Steven N Scott L Middleton MR Wilson RH Mulligan E Curtin N Wang D Dewji R A phase II study of the potent PARP inhibitor, rucaparib (PF-01367338, AG014699), with temozolomide in patients with metastatic melanoma demonstrating evidence of chemopotentiation Cancer Chemother Pharmacol 2013 71 5 1191 1199 10.1007/s00280-013-2113-1 23423489

[B20] Murai J Huang SY Das BB Renaud A Zhang Y Doroshow JH Ji J Takeda S Pommier Y Trapping of PARP1 and PARP2 by clinical PARP inhibitors Cancer Res 2012 72 21 5588 5599 10.1158/0008-5472.CAN-12-2753 23118055 PMC3528345

[B21] Fojo T Bates S Mechanisms of resistance to PARP inhibitors–three and counting Cancer Discov 2013 3 1 20 23 10.1158/2159-8290.CD-12-0514 23319766 PMC6936267

